# 한국 대학병원 옴 진단 환자의 역학적 특성 분석: 단일기관 후향적 연구

**DOI:** 10.7586/jkbns.24.044

**Published:** 2025-02-24

**Authors:** Hye Eun Hwang, Jae Sim Jeong, Yang Ree Kim, Ji young Lee

**Affiliations:** 1 College of Medicine, University of Ulsan, Ulsan, Korea; 1 울산대학교 의과대학; 2 Department of Clinical Nursing, Graduate School of Industry, University of Ulsan, Ulsan, Korea; 2 울산대학교 산업대학원; 3 Division of Infectious Diseases, Department of Internal Medicine, The Catholic University of Korea, Uijeongbu St. Mary’s Hospital, Uijeongbu, Korea; 3 가톨릭대학교 의정부성모병원 감염내과; 4 Infection Control Team, The Catholic University of Korea, Seoul St. Mary Hospital, Seoul, Korea; 4 가톨릭대학교 서울성모병원 감염관리팀

**Keywords:** Scabies, Outpatients, Infection control, 옴, 외래환자, 감염 관리

## Abstract

**Purpose:**

This study aimed to establish infection control strategies for preventing the spread of scabies within a single institution by analyzing the epidemiological characteristics of patients diagnosed with scabies.

**Methods:**

This was a retrospective descriptive study. The electronic medical records of 430 patients diagnosed with scabies at the dermatology outpatient department of Uijeongbu St. Mary’s Hospital from January 2020 to December 2022 were used to collect data on their characteristics. The subjects were divided into three groups: patients, family of confirmed patients, and healthcare workers. General and epidemiological characteristics were analyzed.

**Results:**

The average age was 60.89 ± 22.39 years. The number of days from symptom onset to diagnosis was unknown in many cases (65.3%), although the average was 63.42 ± 64.18 days. Repeated visits after treatment were observed in 193 patients (67.5%), 38 family members of confirmed patients (55.1%), and 35 healthcare workers (46.7%), showing a statistically significant difference (*p* = .002). The most common place of residence before the visit was home (56.2%), and the most common suspected origin of contagion was the home (38.3%), and the most common category of the suspected contagious person was family (49.6%).

**Conclusion:**

Cases of scabies were disproportionately common in women and older adults. The interval from symptom onset to diagnosis was long, and about half of the cases involved itching but no skin lesions. More than one-thirds of cases did not revisit for a follow-up after 2 weeks. In the overall results, unlike previous studies, factors related to home and family were frequently observed as epidemiological characteristics.

## 서론

### 1. 연구의 필요성

옴(scabies)은 옴 진드기(*Sarcoptes scabiei* var. *hominis*)의 피부 기생에 의하여 발생하는 전염성 피부질환으로 접촉이 잦은 요양시설이나 교정시설, 보육시설과 같은 집단 시설에서 주로 발생한다[1]. 국내 위생환경이 좋아지면서 감소하였으나, 최근 인구 고령화와 더불어 요양병원을 중심으로 옴의 발병률이 증가하면서 새로운 공중보건 문제로 대두되고 있다[2]. 해외에서도 인구의 고령화와 요양시설의 장기체류 환자가 늘어나면서 옴 감염이 증가하는 것으로 추정하고 있으며[3], 또한 2020년 coronavirus disease 2019 (COVID-19) 팬데믹 발생 이후 옴의 유병률 증가가 보고되고 있다[4]. 건강보험심사평가원 보건의료 빅데이터 개방시스템의 질병/행위별 의료 통계[5]에서 옴 상병코드(B86)로 조회한 결과 국내에서는 2010년부터 매년 4만여명의 옴 감염자가 확인되고 있다.

세계보건기구는 옴에 대한 인식을 높이고 예방을 위해 2017년에 옴을 방치된 열대성 질병(neglected tropical diseases)으로 공식 지정하고 세계적 통제를 위한 로드맵의 일부에 포함시켰으며, 국제옴통제연합(International Alliance for Control of Scabies, IACS)에서도 옴의 퇴치를 위해 지속적으로 노력하고 있다[6].

전 세계적으로 옴 감염이 증가함에 따라 효과적인 옴 전파 차단을 위해서는 옴 감염에 변화하는 특성이 있는지 지속적으로 모니터링하여 그에 따른 전파방지 대책을 수립하여야 한다. 국내에서 옴은 법정감염병으로 포함되어 있지 않아 발생 여부를 보건소에 신고할 법적인 의무가 없어 환자의 발생 빈도나 변화하는 역학을 파악하기 어려운 실정이다.

또한 옴은 진단의 특성상 임상의사의 주관적인 판단이 필요하므로, 위양성 또는 위음성의 부정확한 진단 가능성이 있다. 특히 비특이적인 옴 증상만이 있는 경우, 가려움 등의 자각적인 증상의 판별이 어려운 경우 진단 지연이 발생할 수 있다[7]. 단일기관에서 옴 역학을 조사한 선행연구[8]에서는 옴을 진단받은 환자 중 45%가 다른 의료기관에서 습진, 피부염 등으로 오진 후 옴으로 진단되었다. IACS 옴 진단 기준[9]에서는 표준화되지 않은 진단방법으로 인해 임상 연구 및 역학조사 결과의 해석에 방해가 될 수 있으므로 옴 유병률 조사 시 대상 인구 특성, 진단 확실성 수준에 따라 접근하는 것을 제시하고 있으나 국내에서는 이와 관련된 연구가 미비하거나 부족한 상황이다. 국외에서는 옴의 유병률과 위험요인에 대해 메타분석한 선행 연구[10]가 있었으며 해당 연구에서는 IACS 옴 진단 기준을 사용할 경우 진단 민감도가 높았고, 위험요인으로 지역, 소득 상태, 연령 등이 통계적으로 유의한 차이를 보였다. 미얀마에서 시행한 연구[11]에서는 나이와 성별, 가족력, 계절 등이 옴 감염과 연관이 있는 것으로 확인되었으며, 스페인에서 시행한 연구[12]에서는 나이, 직업, 감염 장소, 거주 상태 등 위험요인을 다양한 관점에서 조사하여, 의료 환경과 사회적 환경이 주요인임을 확인하였다.

국내 의료환경의 특성에 맞게 옴 감염의 위험요인에 대해 조사한 연구는 제한적이었으므로, 본 연구에서는 옴 진단 환자의 특성을 확인하고, 대상자 분류(일반환자, 의료종사자, 접촉자 가족)에 따른 역학적 특성을 비교함으로써 단일기관 내 유입 또는 전파와 관련된 감염관리 전략을 수립하는데 활용하고자 한다.

### 2. 연구의 목적

본 연구는 최근 3년간 경기 북부지역 소재 700병상 규모의 일 대학병원의 피부과 외래에서 옴을 진단받은 환자들의 특성을 분석하는 후향적 조사연구로 구체적인 목적은 다음과 같다.

첫째, 옴 진단 환자들을 일반환자, 의료종사자, 접촉자 가족으로 분류하여 임상적 특성을 분석한다.

둘째, 옴 진단 환자들의 연도별 월별 발생 빈도와 역학적 특성을 분석한다.

## 연구 방법

### 1. 연구 설계

국내 일개 대학병원에서 진단받은 옴 환자의 특성을 분석하는 후향적 기술역학연구이다.

### 2. 연구 대상

2020년 1월 1일부터 2022년 12월 31일까지 3년간 가톨릭대학교 의정부성모병원 피부과 외래에 내원하여 조직검사 또는 피부 긁음 현미경검사(microscopic test by skin scraping) 등을 통해 옴 벌레 또는 충란을 관찰하거나, 옴 벌레나 충란을 발견하지 못했더라도 임상적으로 진단하여 옴 치료를 한 경우를 포함하였다. 자료 수집 기간 내 진단을 받은 모든 대상자는 누락 없이 포함되었으며, 총 430명이 연구에 포함되었다. 대상자 구분은 노출 경로와 감염 위험도를 고려하여 일반환자, 의료 종사자, 확진자 가족으로 구분하였으며, 의무기록을 기반으로 진행되었다. 연구 기간 내 진단을 받은 전원을 후향적으로 분석하였기 때문에 표본 크기 산출이나 검정력 분석은 수행하지 않았으며, 연구의 주된 목적은 진단을 받은 전체 모집단의 특성을 기술적으로 분석하는데 중점을 두었다.

### 3. 연구 도구

문헌고찰을 통해 옴 진단을 받은 환자의 일반적, 진단적, 치료적, 역학적 특성을 포함하는 증례기록서(Case Report Form)를 개발하였다.

#### 1) 일반적 특성

최초 진단일 기준으로 작성하였으며, 나이, 성별, 진단일, 대상자 종류를 포함하였다. 대상자 종류는 일반환자, 의료종사자, 확진자 가족으로 구분하였다. 내원일 기준 2개월 이내의 의무기록을 포함하여 조사하였고, 외래환자는 치료 후 재진 시 추가적으로 정보가 얻어지는 경우 해당 내용을 포함하여 기록하였다.

기저질환의 세부 구분은 옴의 위험요인에 대한 2011년도의 선행연구[13]를 참조하였고, 당뇨병, 신부전, 간질환, 암, 뇌혈관질환, 만성폐쇄폐질환, 투석, 심장병, 면역질환, 치매, 피부염, 기타로 구분하였다.

#### 2) 진단적 특성

검사, 증상과 징후, 과거 옴 진단 이력를 포함하였다.

(1) 검사: 확진을 위한 검사 시행 유무 및 결과

(2) 증상과 징후: 가려움, 피부 병변 등의 증상 유무와 증상이 있을 경우 증상의 특징과 양상 및 위치, 발현일, 진단 소요기간이다.

(3) 과거 옴 진단 이력: 과거 옴을 진단받아 치료하였으며, 완치된 이력(증상이 지속되어 추가로 진료를 본 경우는 해당하지 않는다)이 있으나 다시 진단받은 경우이다. 본 연구에서는 옴의 최장 잠복기인 4∼6주를 고려하여 2개월 이내에 대한 자료를 수집하였다.

#### 3) 치료적 특성

(1) 치료 약제: 일반적으로 옴 치료에 사용하는 약제들 중 대상자들에게 처방된 약제를 기록하였고, 처방 기간을 포함한다.

(2) 치료(약 2주) 후 재진료 여부: 치료 약제 종류별 용법에 따라 차이가 있으나 약제 도포는 일반적으로 2주간 시행하고 있으며, 치료효과의 확인을 위해 약2∼4주 후 재진을 시행하므로 치료 후 2주 이내에 다시 진료를 받았는지와 재진료 시 임상증상 호전 여부를 포함하였다.

#### 4) 역학적 특성

진료 시 환자 진술에 기반하여 차트에 입력된 정보를 바탕으로 내원 전 거주지, 노출 추정 장소와 추정되는 감염원이다.

(1) 내원 전 거주지: 집, 병원, 요양병원, 요양원, 기타 공동주거시설, 알수없음으로 구분하였다.

(2) 노출 추정 장소: 집, 병원, 요양병원, 요양원, 기타 공동주거시설, 알수없음으로 구분하였다.

(3) 추정되는 감염원: 노출 추정장소에 따라 가족, 입원환자, 의료종사자, 공동거주자, 기타 등으로 구분하였다.

### 4. 자료 수집

본 연구의 자료 수집 기간은 2024년 1월 1일부터 1월 31일까지였으며, 연구기관 Clinical Data Warehouse에서 연구 계획을 승인받은 후 익명화 차트 조회 데이터를 추출하였고, 검색 필터 기능 중 ‘진단정보’ 항목을 사용하여 2020년 1월 1일부터 2022년 12월 31일까지 검사 양성으로 확진 또는 임상적으로 옴이 의심되어 상병코드(B86)가 있는 환자들을 조회하였다. 자료 수집 기간은 COVID-19 팬데믹의 초반부터 안정세를 보이기 시작한 시점까지 포함되어 있으며, 2023년은 백신 보급률이 높아지고 대응 능력이 향상되어 국제보건위기경보의 해제[14] 및 방역 조치가 완화되는 등 상대적으로 COVID-19의 직접적인 영향이 적었으므로, 연구의 일관성을 위해 2023년은 자료 수집 기간에 포함하지 않았다. 확인된 대상자의 의무기록을 검토하여 성별, 연령, 진단 시기, 과거력, 피부 병변의 양상 및 위치, 내원 전 거주지 및 의심되는 감염원, 증상 발현으로부터 진단 소요기간 등의 특성을 확인하였다.

### 5. 자료 분석

수집된 자료는 SPSS version 25.0 (IBM Corp., Armonk, NY, USA) 프로그램을 이용하여 분석하였다. 연구 대상자의 특성은 빈도와 백분율, 평균과 표준편차를 이용하여 분석하였으며, 옴 진단 시기별 분포는 빈도와 백분율로 분석하였다. 대상자 그룹별 진단적, 치료적, 역학적 특성의 차이는 범주형 변수는 chi-square test 또는 Fisher’s exact test로, 연속형 변수는 Kruskal-Wallis test로 분석하고 사후검증은 Bonferroni test로 확인하였다. 모든 통계적 유의수준은 *p* < .05를 기준으로 하였다.

### 6. 윤리적 고려

본 연구는 가톨릭대학교 의정부성모병원 임상연구심의위원회의 심의와 승인(IRB 승인번호: UC23WASI0133) 후 자료를 수집하였다. 후향적 연구로서 진료과정에서 확보된 의무기록을 활용하며, 분석 과정에서 특정이 가능한 정보는 익명화하였다. 연구를 통해 확보된 의무기록은 파일형태로 암호화하여 연구자의 개인 노트북에 보관하였으며, 연구자 외에는 접근하거나 사용할 수 없도록 하였다. 승인된 기간 동안 연구 목적으로만 사용하며, 연구 종료 후에는 신뢰성 확보를 위하여 3년간 보관한 후 복원하지 못하도록 파일을 삭제할 것이다.

## 연구 결과

### 1. 일반적 특성

전체 430명의 대상자는 각 군 모두 여성이 많았으며, 일반환자 157명(54.9%), 확진자 가족 40명(58.0%), 의료종사자 58명(77.3%)으로 통계적으로 유의한 차이를 나타내었다(*p* = .002). 일반환자에서는 80세 이상이 96명(33.6%)으로 가장 많았고, 확진자 가족은 40세 미만이 21명(30.4%), 의료종사자는 60∼69세가 30명(40.0%)으로 가장 많았고, 대상자 그룹별 평균 연령은 일반환자 66.35 ± 21.43세, 확진자 가족 45.86 ± 24.92세, 의료종사자 53.88 ± 13.82세로 통계적으로 유의한 차이를 나타내었다(*p* < .001). 기저질환은 고혈압 106명(55.5%), 당뇨병 92명(48.2%), 치매 46명(24.1%) 순으로 많았으며 대상자 그룹 간 통계적으로 유의한 차이를 나타내지는 않았다(Table 1).

### 2. 진단적 특성

전체 대상자 중 피부 긁음 현미경검사 양성인 경우 204명(47.4%), 음성인 경우가 172명(40.0%), 시행하지 않은 경우는 54명(12.6%)이었다. 대상자 그룹별 양성자는 일반환자 166명(58.1%), 확진자 가족 18명(26.1%), 의료종사자 20명(26.7%)으로 통계적으로 유의한 차이를 보였다(*p* < .001). 전체 대상자 중 증상이 있는 경우는 404명(94.0%)으로, 일반환자에서 증상이 있는 경우가 280명(97.9%)으로 가장 많았으며 확진자 가족 57명(82.6%), 의료종사자 67명(89.3%)으로 통계적으로 유의한 차이를 보였다(*p* < .001). 증상이 있는 대상자 중 가려움이 있는 경우는 372명(92.1%)이었으며, 야간에 특징적인 가려움을 호소한 경우는 18명(4.8%)이었다. 피부 병변이 있는 경우는 222명(55.0%)이었으며, 구진(papule)과 판(plaque)이 151명(68.0%)으로 가장 많이 관찰되었다. 피부 병변의 유무와 종류에서 대상자 그룹 간에 통계적으로 유의한 차이를 보이지는 않았다. 호발 부위는 전신이 105명(47.3%)으로 가장 많았고, 상체에서 일반환자 13명(8.1%), 확진자 가족 7명(29.2%), 의료종사자 5명(13.5%)으로 통계적으로 유의한 차이를 보였다(*p* = .012). 증상 발현일로부터 진단까지 소요 기간은 알 수 없는 경우가 264명(65.3%)으로 가장 많았으며, 평균 63.42 ± 64.18일이 소요되었다. 대상자 그룹별 진단까지의 소요 기간은 일반환자 64.36 ± 61.82일, 확진자 가족 54.71 ± 49.59일, 의료종사자 65.38 ± 77.83일로 통계적으로 유의한 차이를 보이지는 않았다. 2개월 이내 옴 진단이력이 있는 경우는 21명(4.9%)으로 대상자 그룹 간 통계적으로 유의한 차이를 보이지는 않았다(Table 2).

### 3. 치료적 특성

치료 연고는 전체 430명 중 352명(81.9%)에게 처방되었으며, permethidine이 303명(86.1%), crotamiton이 49명(13.9%) 순이었다. 전체 치료 후 재진 환자는 총 266명(61.9%)이었으며, 방문하지 않은 경우는 164명(38.1%)이었다. 치료 후 재진 여부는 일반환자, 확진자 가족, 의료종사자 간에 유의한 차이가 있었고(*p* = .002), 재진 환자는 일반환자가 193명(67.5%)으로 확진자 가족 38명(55.1%), 의료종사자 35명(46.7%)에 비해 많았다. 재진 시 증상이 없는 경우는 93명(35.0%), 일부 완화되었지만 증상이 남아있는 경우 130명(48.9%), 호전되지 않거나 악화된 경우가 43명(16.1%)이었다(Table 3).

### 4. 역학적 특성

#### 1) 시기별 환자 분포

**Figure 1.**
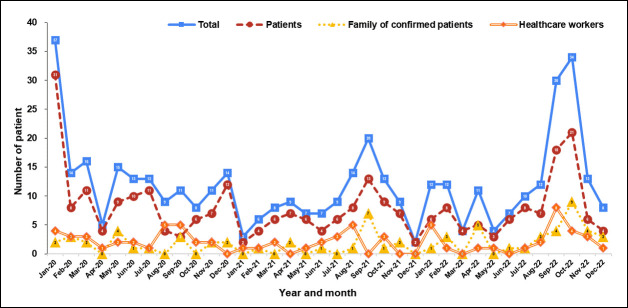
Scabies patient distribution by year and month.

430명의 환자의 연도별 진단 수는 2020년 166명, 2021년 107명, 2022년 157명으로 감소했다가 증가하는 추이를 보였다. 2020년에는 1월에 많이 발생하였고 2021년에는 8∼10월, 2022년에는 9∼10월에 많이 발생한 경향을 나타내었다(Figure 1).

#### 2) 전파와 관련된 특성

내원 전 거주지에 대한 기록 유무는 일반환자, 확진자 가족, 의료종사자 간에 유의한 차이가 있었고(*p* < .001), 확진자 가족은 69명(100.0%), 의료종사자 75명(100.0%)으로 일반환자 196명(68.5%)에 비하여 많았다. 내원 전 거주지는 집인 경우가 191명(56.2%)으로 가장 많았으며, 대상자 그룹별로 유의한 차이가 있었다(*p* < .001). 일반환자는 요양기관이 107명(54.6%)으로 가장 많았으며, 확진자 가족은 67명(97.1%), 의료종사자는 60명(80.0%)으로 집이 가장 많았다.

의심되는 감염 장소에 대한 기록 유무는 일반환자, 확진자 가족, 의료종사자 간에 유의한 차이가 있었고(*p* < .001), 확진자 가족은 69명(100.0%), 의료종사자 70명(93.3%)으로 일반환자 36명(12.6%)에 비하여 많았다. 의심되는 감염 장소는 대상자 그룹별로 유의한 차이가 있었으며(*p* < .001), 일반환자에서는 요양기관이 16명(44.4%)으로 가장 많았고, 확진자 가족에서는 집이 62명(89.9%)으로 가장 많았으며, 의료종사자에서는 요양기관이 37명(52.9%)으로 가장 많았다.

의심되는 감염대상자에 대한 기록(Records regarding suspected contagious persons)은 일반환자, 확진자 가족, 의료종사자 간에 유의한 차이가 있었고(*p* < .001), 확진자 가족은 69명(100.0%), 의료종사자 35명(46.7%)으로 일반환자 31명(10.8%)에 비하여 많았다. 의심되는 감염원은 대상자 그룹별로 유의한 차이가 있었으며(*p* < .001), 일반환자에서는 요양기관 또는 병원 환자가 13명(41.9%)로 가장 많았고, 확진자 가족에서는 가족이 63명(91.3%)으로 가장 많았으며, 의료종사자에서는 요양기관 환자가 17명(48.6%)으로 가장 많았다.

입원력은 있는 경우가 67명(15.6%), 없는 경우가 363명(84.4%)이었다. 입원력이 있는 경우는 일반환자가 66명(23.1%)로 확진자 가족 1명(1.4%), 의료종사자 0명(0.0%)에 비해 많았으며, 통계적으로 유의한 차이를 보였다(*p* < .001)(Table 4).

## 논의

본 연구는 2020년 1월부터 2022년 12월까지 경기 북부 소재의 가톨릭대학교 의정부성모병원에 내원하여 옴을 진단받은 430명의 대상자의 일반적인 특성 및 임상적 특징, 역학적 특성을 조사하였다. 또한 특성에 차이가 있는 세 대상자 그룹을 비교함으로써 옴 감염 및 전파의 위험 요인을 확인하고, 감염관리 전략 수립에 활용하고자 하였다.

전체 430명의 환자의 연도별 진단 수는 2020년 166명, 2021년 107명, 2022년 157명으로 2021년에 감소했다가 증가하는 추이를 보였으며, 월별 분포는 2021년과 2022년은 9월과 10월에서 많은 것으로 나타났다. 연구자가 국내 보험심사평가원 보건의료 빅데이터 개방시스템의 질병/행위별 의료 통계[5]에서 3년 간의 옴 진단 환자를 연령을 구분하여 상병코드(B86)로 조회한 결과 9∼11월에 많이 발생한 것으로 나타나 본 연구와 유사한 추이를 보였다. 2020년 COVID-19 팬데믹 발생 이후 국외에서는 옴의 유병률 증가가 보고되고 있으나 국외와 다르게 국내 및 본 연구에서 옴이 증가하지 않은 것에 대해서는 추가적인 연구가 필요하다.

전체 대상자 중 404명(94.0%)이 증상을 가지고 있었으며, 증상이 있는 전체 대상자 중 가려움이 있는 경우는 372명(92.1%), 피부 병변이 있는 경우는 222명(55.0%)이었다. 가려움의 유무와 발생 시간, 피부 병변의 유무에서 대상자 그룹 간에 통계적으로 유의한 차이는 없었다. 전형적인 피부 병변 없이 가려움만 있는 경우에도 옴으로 진단받을 수 있도록 의료기관 및 지역사회 내 홍보가 필요할 것으로 생각한다.

대상자의 증상 발현일로부터 진단까지는 평균 63.42 ± 64.18일이 소요되었으며, 세 그룹 간에 통계적으로 유의한 차이는 없었다. 조사 대상자의 의무기록 중에는 가려움이 있었으나 진료를 보지 않고 약국에서 연고를 구입한 후 악화되자 내원한 경우도 있었다. 확진자 가족이나 의료종사자의 경우 옴에 대한 인식이 있는 상태였을 가능성이 높았음에도 대상자 그룹 모두에서 평균 진단 소요기간이 긴 것은 전형적인 야간의 가려움이나 피부 병변 없이 비특이적인 증상만 있는 경우가 많아 병원에 내원하지 않거나, 내원하였더라도 진단에 어려움이 있었을 것으로 추측한다. 대상자가 내원하지 않거나 오진으로 진단되지 않을 경우 일상생활을 하며 주변인에게 전염시키는 등 지역사회에서 잠재적인 감염원으로 작용할 수 있으므로[15] 이에 대한 효과적인 감염관리 전략의 수립이 필요하다.

치료 후 다시 진료받은 경우는 일반환자 193명(67.5%), 확진자 가족 38명(55.1%), 의료종사자 35명(46.7%)로 차이가 있었다. 재진 시 증상이 없어 치료가 완료된 경우는 일반환자 63명(32.6%), 확진자 가족 11명(28.9%), 의료종사자 19명(54.3%)로 통계적으로 유의한 차이를 보이지는 않았다. 의료종사자의 치료 후 재진율이 낮은 이유는 재진 시 증상이 없어 치료가 완료된 경우가 높았던 것과 관련하여 다른 대상자 그룹에 비해 치료연고 도포 방법이나 주변환경 관리 등이 적절하여 치료가 성공하였고, 추가적인 진료가 필요하지 않았을 가능성이 있다.

본 연구에서는 2주 간의 치료 기간 후 호전되지 않은 경우와 관련하여 연고 도포 등이 부적절하였는지 또는 기타 요인으로 인한 것인지는 명확하게 알 수 없으므로 이에 대한 후속 연구가 필요할 것으로 생각한다. 20여개의 연구에서 옴 치료 실패와 관련이 있는 것으로 추정되는 요인을 메타분석한 선행연구[16]에서는 14일 추적 관찰 후 치료 실패율을 15.2%로 보고하고 있으며, 이는 본 연구보다는 낮은 수치에 해당한다. 또한 해당 연구에서는 치료 실패요인을 약물의 효능 및 저항력, 부적절한 적용, 치료 지연, 인지, 행동 또는 이동장애(노화 또는 기타 요인으로 인해), 노화된 피부의 표면 지질 함량 손상 등으로 제시하고 있다. 프랑스에서 옴 치료 실패 요인 추적을 위해 시행된 선행연구[17]에서는 치료실패 요인으로 밀접접촉자에 대한 불충분한 관리가 42%로 나타났으며, 부적절한 치료 방법의 적용이 48%로 나타났다. 특히 연고를 적용하고 손을 씻음으로써 손에는 적절한 연고 적용이 이루어지지 않는 부분이 확인되어 정확하고 반복적인 설명이 필요함을 언급하였다.

선행연구에서 확인된 것처럼 부적절한 치료 방법의 적용을 줄이기 위해 연고 도포방법 교육에 대한 현행 파악 및 환자의 치료 순응도를 높일 수 있는 교육 전략을 수립하는 것이 필요하다. 독일에서 시행된 선행 연구[18]에 따르면 서면화된 정보를 제공하지 않을 경우 치료 실패율이 높았다. 연구 대상 기관에서의 옴 진단환자에 대한 설명 방법을 확인한 결과 진료 후 서면화된 안내문을 제공하고 설명하고 있었으나, 연령이나 대상자에 관계없이 동일한 내용을 제공하고 있었다. 따라서 대상자 특성에 따라 적합한 안내문을 개발하고 그에 대한 효과를 평가할 것이 필요하다.

옴 진단환자는 2주간의 치료 후 재진을 통해 경과를 관찰할 것을 권고하고 있으나, 38.1%의 환자는 내원하지 않았다. 재진일자에 방문하지 않은 환자는 추적할 수 없으므로 해당환자들이 증상이 호전되어 내원하지 않았는지, 호전되지 않았음에도 방문하지 않았는지 알 수 없었다. 프랑스에서 시행한 선행 연구[17]에서는 2주 후 치료를 받지 않은 환자의 수가 많았으며 이는 옴 치료 실패의 원인 중 하나로 지적되었다. 지역사회 내 숨은 전파를 방지하기 위해서는 진단의 확실성이 낮은 환자도 치료를 완료할 수 있도록 강화된 설명이 필요하다.

내원 전 거주지는 집이 191명(56.2%)으로 가장 많았으며, 의심되는 감염 장소 역시 집이 67명(38.3%)으로 가장 많았고 의심되는 감염원은 가족이 67명(49.6%)으로 가장 많았으나, 대상자 그룹 간 비교한 결과 확진자 가족을 제외한 두 그룹에서는 요양기관과 관련된 요인이 높게 확인되었다. 의심되는 감염장소에서 요양기관은 일반환자 16명(44.4%), 의료종사자 37명(52.9%)으로 높게 나타났으며, 의심되는 감염원은 요양기관 환자가 일반환자 13명(41.9%), 의료종사자 17명(48.6%)으로 높게 나타났다. 2013년에 시행된 국내 연구[19]에서는 의심되는 감염 장소가 자택에서 높게 나타났으나, 이후 시행된 연구들에서는 요양기관과 관련된 요인이 높게 나타나 본 연구와 유사하였다. 2019년과 2020년에 시행된 국내 연구[20,21]와 국내 경기 서북부지역의 대학병원에서 시행된 선행 연구[22]에서는 장기요양기관과 관련된 요인이 의심되는 감염원으로 높게 보고되었다. 본 연구에서는 의무기록 상의 정보가 불충분하여 역학적 특성 항목의 내원 전 거주지, 의심되는 감염장소, 의심되는 감염원에서 알 수 없음의 비율이 높았고, 확진자 가족을 제외한 두 군에서는 요양기관과 관련된 요인이 높았던 점을 고려하면 본 연구에서 요양기관과 관련된 요인이 실제보다 적게 나타났을 가능성이 있다. 따라서 변화되는 역학적 특성 확인과 적절한 감염관리 전략 수립을 위해서는 진료 시 대상자에 대한 강화된 기록이 필요할 것으로 보인다. 또한 지역 내 옴 유행사례를 다룬 이탈리아의 연구[23]에서는 가족과 관련된 요인을 통제함으로써 지역사회 내 병원과 요양기관의 전파를 막는데 효과적이었다는 보고가 있다. 본 연구에서 가족과 관련된 요인이 높게 나타났던 것과 관련하여, 가족과 관련된 전파 통제 전략을 수립하고 그 효과에 대해 평가하는 연구가 필요할 것으로 보인다.

본 연구에서는 COVID-19 팬데믹 기간 동안 옴 진단 건 수가 유의하게 증가하지는 않았으나, 유행으로 추정되는 시기가 있었다. 미국 CDC의 성병 감시보고서[24]에 따르면 매독 역시 COVID-19 팬데믹 기간 동안 증가하였고, 공중보건 비상사태에 따라 한정된 인력과 자원이 집중되면서 전파에 대한 조기 통제가 이루어지지 않은 것으로 추정하고 있다. 스페인에서 시행된 선행 연구[25]에서는 COVID-19 팬데믹 격리기간 동안 옴 사례가 크게 증가하였으며, 특히 가족이나 동거인이 옴으로 진단된 환자에서 진단되는 경우가 유의하게 많았다. 증상 기간 또한 격리하지 않은 경우에 비해 유의하게 증가하였다. 격리로 인해 각 가족 집단 내에서 경미한 집단 발생이 있었음을 추정하였으며, 의료기관 방문에 대한 두려움이 전염을 증가시킨 것으로 추정하고 있다. 대규모 전염병의 발생 시 의료 접근성 약화로 인해 밀접접촉을 통해 전파되는 질병이 증가할 수 있으며, 지역사회 내 적절한 차단 조치를 위해서는 그에 대한 의료 자원 배치 전략 수립이 필요할 것으로 보인다.

본 연구는 옴 감염에 대해 진단, 치료 및 생리적 특성을 포괄적으로 조사함으로써 예방 및 관리에 활용할 수 있는 기초자료를 제공하며, 이를 통해 간호 실무의 정확성과 효율성을 향상시키고, 감염관리 정책에 반영할 수 있다는 기초간호학적 의의가 있다. 본 연구의 제한점은 첫째, 후향적으로 환자의 의무기록에 의존하였으므로 진료기록이 부정확하여 자료가 왜곡되었을 가능성이 있다. 둘째, 옴은 진료의의 임상적인 판단으로도 진단이 가능하므로, 실제적인 옴 환자가 아니었을 가능성도 있다. 셋째, 단일기관에 한정하여 진행한 연구이므로 다른 지역 또는 기관에 확대하여 적용하기에는 무리가 있다. 선행연구와 차이를 보였던 부분은 지역적인 특성을 고려하여 여러 규모의 병원을 대상으로 연구가 필요하다.

## 결론

국외 선행연구와 달리 COVID-19 팬데믹 기간 중 옴 진단환자가 크게 증가하지는 않았으나, 유행으로 의심되는 기간이 있었다. 일반환자, 확진자 가족, 의료종사자 모두 고령자가 많았다. 증상발현일로부터 진단에 소요되는 기간도 길었으며, 가려움은 있으나 피부 병변이 없는 경우도 약 반을 차지하였다. 2주 후 재진 방문을 하지 않는 경우는 1/3 이상이었으며, 재진 환자 중 증상이 남아있거나 악화되는 경우도 절반 이상을 차지하였다. 국내 선행연구와 달리 의심되는 감염장소가 집이거나 의심되는 감염원이 가족인 경우가 가장 많았으나, 확진자 가족을 제외한 두 그룹에서는 요양기관과 관련된 요인이 높게 확인되었다. 본 연구는 경기 북부 의료기관 및 지역사회에서 옴과 관련된 위험 요인을 추정한 연구로서, 본 연구에서 조사된 옴 감염환자의 특성에 따라 교육 계획을 수립하고 그를 통한 효과 평가를 시행하는 연구를 제언한다. 또한 본 연구는 단일 대학병원에 한하여 의무기록을 통하여 후향적인 조사를 실시하였으므로, 보편적인 감염관리 전략 수립을 위해서는 추후 여러 규모의 의료기관을 대상으로 전향적인 조사를 실시하여 연구할 것을 제언한다.

## Figures and Tables

**Table 1.** General Characteristics of Participants Diagnosed with Scabies (N = 430)

Variables	Categories	Total	Classification			χ² or H	*p*
			Patients^a^ (n = 286)	Family of confirmed patients^b^ (n = 69)	Healthcare workers^c^ (n = 75)		
		n (%) or M ± SD					
Sex	Women	255 (59.3)	157 (54.9)	40 (58.0)	58 (77.3)	12.46	.002
	Men	175 (40.7)	129 (45.1)	29 (42.0)	17 (22.7)		
Age (yr)	< 40	64 (14.9)	30 (10.5)	21 (30.4)	13 (17.3)	97.16	< .001
	40~49	36 (8.4)	16 (5.6)	12 (17.4)	8 (10.7)		
	50~59	78 (18.1)	41 (14.3)	16 (23.2)	21 (28.0)		
	60~69	87 (20.2)	49 (17.1)	8 (11.6)	30 (40.0)		
	70~79	62 (14.4)	54 (18.9)	6 (8.7)	2 (2.7)		
	≥ 80	103 (24.0)	96 (33.6)	6 (8.7)	1 (1.3)		
	Total	60.89 ± 22.39	66.35 ± 21.43	45.86 ± 24.92	53.88 ± 13.82	65.53^†^	< .001
							(a > b,c)
Presence of underlying diseases	Yes	191 (44.4)	164 (57.3)	15 (21.7)	12 (16.0)	58.26	< .001
	No	239 (55.6)	122 (42.7)	54 (78.3)	63 (84.0)		
Underlying diseases (n = 191, multiple choices)	Hypertension	106 (55.5)	88 (53.7)	10 (66.7)	8 (66.7)	1.59	.452
	Diabetes	92 (48.2)	82 (50.0)	4 (26.7)	6 (50.0)	3.02	.222
	Dementia	46 (24.1)	44 (26.8)	1 (6.7)	1 (8.3)	4.79	.091
	Cerebrovascular disease	39 (20.4)	38 (23.2)	1 (6.7)	0 (0.0)	5.59	.061
	Heart disease	35 (18.3)	31 (18.9)	3 (20.0)	1 (8.3)	0.87	.649
	Renal failure	27 (14.1)	24 (14.6)	2 (13.3)	1 (8.3)	0.37	.829
	Dialysis	12 (6.3)	12 (7.3)	0 (0.0)	0 (0.0)	2.11	.349
	Cancer	10 (5.2)	7 (4.3)	2 (13.3)	1 (8.3)	2.52	.283
	Liver disease	8 (4.2)	8 (4.9)	0 (0.0)	0 (0.0)	1.38	.503
	COPD	5 (2.6)	5 (3.0)	0 (0.0)	0 (0.0)	0.85	.655
	Others	25 (13.1)	23 (14.0)	1 (6.7)	1 (8.3)	0.91	.635

M = Mean; SD = Standard deviation; COPD = Chronic obstructive pulmonary disease.

† Kruskal-Wallis test with the Bonferroni correction for pairwise comparisons.

**Table 2.** Diagnostic Characteristics and Signs and Symptoms of Participants (N = 430)

Variables	Categories	Total	Classification			χ² or H	*p*
			Patients^a^ (n = 286)	Family of confirmed patients^b^ (n = 69)	Healthcare workers^c^ (n = 75)		
		n (%) or M ± SD					
Microscopic test by skin scraping	Positive	204 (47.4)	166 (58.1)	18 (26.1)	20 (26.7)	43.07	<.001
	Negative	172 (40.0)	85 (29.7)	41 (59.4)	46 (61.3)		
	No result	54 (12.6)	35 (12.2)	10 (14.5)	9 (12.0)		
Presence of signs and symptoms	Yes	404 (94.0)	280 (97.9)	57 (82.6)	67 (89.3)	26.30	<.001
	No	26 (6.0)	6 (2.1)	12 (17.4)	8 (10.7)		
Pruritis (n = 404)	Yes	372 (92.1)	260 (92.9)	53 (93.0)	59 (88.1)	1.78	.411
	No	32 (7.9)	20 (7.1)	4 (7.0)	8 (11.9)		
Type of pruritis (n = 372)	No specific time	354 (95.2)	247 (95.0)	49 (92.5)	58 (98.3)	2.13	.346
	Severe at night	18 (4.8)	13 (5.0)	4 (7.5)	1 (1.7)		
Skin lesion (n = 404)	Yes	222 (55.0)	161 (57.5)	24 (42.1)	37 (55.2)	4.54	.103
	No	182 (45.0)	119 (42.5)	33 (57.9)	30 (44.8)		
Type of skin lesion (n = 222, multiple choices)	Papule, plaque	151 (68.0)	106 (65.8)	15 (62.5)	30 (81.1)	3.59	.166
	Burrow	26 (11.7)	19 (11.8)	2 (8.3)	5 (13.5)	0.38	.826
	Wheal, rash	20 (9.0)	18 (11.2)	1 (4.2)	1 (2.7)	3.41	.182
	Pustule, vesicle	19 (8.6)	15 (9.3)	4 (16.7)	0 (0.0)	5.60	.061
	Hyperkeratosis	15 (6.8)	13 (8.1)	1 (4.2)	1 (2.7)	1.67	.435
	Nodule	14 (6.3)	10 (6.2)	1 (4.2)	3 (8.1)	0.39	.822
	Macule, patch	12 (5.4)	11 (6.8)	1 (4.2)	0 (0.0)	2.83	.243
	Crust	9 (4.1)	8 (5.0)	1 (4.2)	0 (0.0)	1.91	.385
	Lichenification, scales	3 (1.4)	3 (1.9)	0 (0.0)	0 (0.0)	1.15	.562
	Bulla	3 (1.4)	3 (1.9)	0 (0.0)	0 (0.0)	1.15	.562
	Excoriation, scar	2 (0.9)	2 (1.2)	0 (0.0)	0 (0.0)	0.77	.682
	Others	6 (2.7)	5 (3.1)	1 (4.2)	0 (0.0)	1.32	.516
Site of skin lesion (n = 222, multiple choices)	Whole body	105 (47.3)	81 (50.3)	8 (33.3)	16 (43.2)	2.71	.258
	Hand, foot	35 (15.8)	23 (14.3)	6 (25.0)	6 (16.2)	1.81	.404
	Groin	30 (13.5)	20 (12.4)	6 (25.0)	4 (10.8)	3.11	.212
	Upper body	25 (11.3)	13 (8.1)	7 (29.2)	5 (13.5)	8.87	.012
	Lower body	24 (10.8)	19 (11.8)	3 (12.5)	2 (5.4)	1.36	.508
	Arm, wrist, elbow	17 (7.7)	8 (5.0)	4 (16.7)	5 (13.5)	5.93	.052
	Axilla	5 (2.3)	4 (2.5)	1 (4.2)	0 (0.0)	1.29	.524
	Others	10 (4.5)	6 (3.7)	2 (8.3)	2 (5.4)	1.11	.573
Number of days from symptom onset to diagnosis (n = 404)	0~30	53 (13.1)	32 (11.4)	7 (12.3)	14 (20.9)	14.12	.167
	31~60	30 (7.4)	19 (6.8)	5 (8.8)	6 (9.0)		
	61~90	28 (6.9)	19 (6.8)	2 (3.5)	7 (10.4)		
	91~120	12 (3.0)	11 (3.9)	1 (1.8)	0 (0.0)		
	> 120	17 (4.2)	10 (3.6)	2 (3.5)	5 (7.5)		
	Unknown	264 (65.4)	189 (67.5)	40 (70.1)	35 (52.2)		
	Total	63.42 ± 64.18	64.36 ± 61.82	54.71 ± 49.59	65.38 ± 77.83	0.64^†^	.726
Previous history of Scabies within 2 months	Yes	21 (4.9)	18 (6.3)	2 (2.9)	1 (1.3)	3.85	.146
	No	409 (95.1)	268 (93.7)	67 (97.1)	74 (98.7)		

M = Mean; SD = Standard deviation.

† Kruskal-Wallis test with the Bonferroni correction for pairwise comparisons.

**Table 3.** Therapeutic Characteristics of Participants (N = 430)

Variables	Categories	Total	Classification			χ²	*p*
			Patients (n = 286)	Family of confirmed patients (n = 69)	Healthcare workers (n = 75)		
		n (%)					
Ointment prescription	Yes	352 (81.9)	230 (80.4)	61 (88.4)	61 (81.3)	2.41	.300
	No	78 (18.1)	56 (19.6)	8 (11.6)	14 (18.7)		
Type of ointment (n = 352)	Permethidine	303 (86.1)	200 (87.0)	49 (80.3)	54 (88.5)	2.14	.344
	Crotamiton	49 (13.9)	30 (13.0)	12 (19.7)	7 (11.5)		
	Total	352 (100.0)	230 (100.0)	61 (100.0)	61 (100.0)		
Repeat visit to the outpatient clinic	Yes	266 (61.9)	193 (67.5)	38 (55.1)	35 (46.7)	12.52	.002
	No	164 (38.1)	93 (32.5)	31 (44.9)	40 (53.3)		
Outcome of the repeat visit (n = 266)	Cured and no symptoms	93 (35.0)	63 (32.6)	11 (28.9)	19 (54.3)	7.71	.103
	Improvement with some symptoms remaining	130 (48.9)	97 (50.3)	22 (57.9)	11 (31.4)		
	No improvement or aggravation	43 (16.1)	33 (17.1)	5 (13.2)	5 (14.3)		
	Total	266 (100.0)	193 (100.0)	38 (100.0)	35 (100.0)		

**Table 4.** Transmission-related Characteristics of Participants (N = 430)

Variables	Categories	Total	Classification			χ²	*p*
			Patients (n = 286)	Family of confirmed patients (n = 69)	Healthcare workers (n = 75)		
		n (%)					
Records of residence before visit	Present	340 (79.1)	196 (68.5)	69 (100.0)	75 (100.0)	57.31	< .001
	Not present	90 (20.9)	90 (31.5)	0 (0.0)	0 (0.0)		
Residence before visit (n = 340)	Home	191 (56.2)	64 (32.7)	67 (97.1)	60 (80.0)	144.64	< .001
	Long term care hospital or nursing home	108 (31.8)	107 (54.6)	0 (0.0)	1 (1.3)		
	General hospital	29 (8.5)	13 (6.6)	2 (2.9)	14 (18.7)		
	Others	12 (3.5)	12 (6.1)	0 (0.0)	0 (0.0)		
Records regarding suspected origin of contagion	Present	175 (40.7)	36 (12.6)	69 (100.0)	70 (93.3)	280.28	< .001
	Not present	255 (59.3)	250 (87.4)	0 (0.0)	5 (6.7)		
Suspected origin of contagion (n = 175)	Home	67 (38.3)	4 (11.1)	62 (89.9)	1 (1.4)	174.77	< .001
	Long term care hospital or nursing home	54 (30.9)	16 (44.4)	1 (1.4)	37 (52.9)		
	General hospital	39 (22.2)	3 (8.3)	6 (8.7)	30 (42.9)		
	Others	15 (8.6)	13 (36.2)	0 (0.0)	2 (2.8)		
Records regarding suspected contagious persons	Present	135 (31.4)	31 (10.8)	69 (100.0)	35 (46.7)	215.01	< .001
	Not present	295 (68.6)	255 (89.2)	0 (0.0)	40 (53.3)		
Suspected contagious persons (n = 135)	Family	67 (49.6)	4 (12.9)	63 (91.3)	0 (0.0)	135.75	< .001
	Patients from long term care hospital or nursing home	30 (22.2)	13 (41.9)	0 (0.0)	17 (48.6)		
	Patients from general hospital	18 (13.3)	4 (12.9)	5 (7.2)	9 (25.7)		
	Healthcare worker	11 (8.2)	1 (3.2)	1 (1.5)	9 (25.7)		
	Others	9 (6.7)	9 (29.1)	0 (0.0)	0 (0.0)		
Admission history within 2 months	Yes	67 (15.6)	66 (23.1)	1 (1.4)	0 (0.0)	36.54	< .001
	No	363 (84.4)	220 (76.9)	68 (98.6)	75 (100.0)		

## Data Availability

Please contact the corresponding author for data availability.
